# Genomic Prediction Using Bayesian Regression Models With Global–Local Prior

**DOI:** 10.3389/fgene.2021.628205

**Published:** 2021-04-15

**Authors:** Shaolei Shi, Xiujin Li, Lingzhao Fang, Aoxing Liu, Guosheng Su, Yi Zhang, Basang Luobu, Xiangdong Ding, Shengli Zhang

**Affiliations:** ^1^National Engineering Laboratory for Animal Breeding, College of Animal Science and Technology, China Agricultural University, Beijing, China; ^2^Guangdong Provincial Key Laboratory of Waterfowl Healthy Breeding, College of Animal Science and Technology, Zhongkai University of Agriculture and Engineering, Guangzhou, China; ^3^Medical Research Council Human Genetics Unit, Institute of Genetics and Molecular Medicine, The University of Edinburgh, Edinburgh, United Kingdom; ^4^Center for Quantitative Genetics and Genomics, Aarhus University, Tjele, Denmark; ^5^Shannan Animal Husbandry and Veterinary Master Station, Shannan, China

**Keywords:** half-Cauchy, half-t distribution, Horseshoe+ prior, hyperparameter estimating, Horseshoe

## Abstract

Bayesian regression models are widely used in genomic prediction for various species. By introducing the global parameter τ, which can shrink marker effects to zero, and the local parameter λ_*k*_, which can allow markers with large effects to escape from the shrinkage, we developed two novel Bayesian models, named BayesHP and BayesHE. The BayesHP model uses Horseshoe+ prior, whereas the BayesHE model assumes local parameter λ_*k*_, after a half-t distribution with an unknown degree of freedom. The performances of BayesHP and BayesHE models were compared with three classical prediction models, including GBLUP, BayesA, and BayesB, and BayesU, which also applied global–local prior (Horseshoe prior). To assess model performances for traits with various genetic architectures, simulated data and real data in cattle (milk production, health, and type traits) and mice (type and growth traits) were analyzed. The results of simulation data analysis indicated that models based on global–local priors, including BayesU, BayesHP, and BayesHE, performed better in traits with higher heritability and fewer quantitative trait locus. The results of real data analysis showed that BayesHE was optimal or suboptimal for all traits, whereas BayesHP was not superior to other classical models. For BayesHE, its flexibility to estimate hyperparameter automatically allows the model to be more adaptable to a wider range of traits. The BayesHP model, however, tended to be suitable for traits having major/large quantitative trait locus, given its nature of the “U” type-like shrinkage pattern. Our results suggested that auto-estimate the degree of freedom (e.g., BayesHE) would be a better choice other than increasing the local parameter layers (e.g., BayesHP). In this study, we introduced the global–local prior with unknown hyperparameter to Bayesian regression models for genomic prediction, which can trigger further investigations on model development.

## Introduction

The genomic prediction has been widely applied in animal and plant breeding. The statistical method being used is one of the critical factors for the accuracy of genomic estimated breeding value and consequently impacting the genetic gain achieved by genomic prediction. Models commonly used for predicting genomic estimated breeding value can be divided into two categories: (i) methods based on the framework of best linear unbiased prediction (BLUP), such as GBLUP (Habier et al., 2007; VanRaden, 2008); (ii) a set of Bayesian regression models – such as BayesA and BayesB (Meuwissen et al., 2001), which are also called as “Bayesian alphabet” models (Gianola et al., 2009). GBLUP assumes that the effects of all genetic markers are normally distributed and share the same variance, thus fitting well for traits with polygenic inheritance. In Bayesian methods, the marker effects are relevant to the choice of the prior probability distribution. By giving different priors, the Bayesian models can fit well for traits with different genetic architectures. For example, the widely used BayesA and BayesB models allow effects of genetic markers to follow a heavy-tailed distribution and therefore in line with the real distribution of marker effects for traits with large quantitative trait locus (QTL).

Bayesian regression models can be further classified into a one-group model and a two-group model from the perspective of the number of groups of genetic markers being used when estimating marker effects. The one-group model is generally a variable shrinkage model that shrinks the effects of some markers toward zero, such as BayesA (Meuwissen et al., 2001). The two-group model (or spike-and-slab model), can also be a multigroup model, is generally a variable selection model that only selects a subset of markers to be included in the model and assumes the remaining markers to have zero effects, such as BayesB (Meuwissen et al., 2001) and BayesC (Habier et al., 2011). The shrinkage process and the selection process can also be combined in a Bayesian regression model. In such a model, a subset of markers was selected to be included in the model, and then, the effects of some selected markers were further shrunk toward zero. BayesCπ (Habier et al., 2011) is an example of this type of model. Compared with BayesB and BayesC, BayesCπ can estimate the proportion of genetic markers with a non-zero effect based on the data. However, BayesCπ could be challenged by problems such as poor convergence and mixing in some situations (Wolc et al., 2011).

A Bayesian regression model with “global–local” shrinkage prior could be a good alternative for genomic prediction. With the global–local prior, the variances of marker effects can be shaped by global and local parameters simultaneously. The global parameter,τ, can shrink the marker effects to approach zero, whereas the local parameter, λ_*k*_, allows a marker to escape from the shrinkage when the marker has a big effect (Piironen and Vehtari, 2017). Horseshoe prior (Carvalho et al., 2010) is one of the most popular estimators of global–local prior. In Horseshoe prior, the local parameter follows a positive half-Cauchy distribution, which is a special case of half-t distribution where the degree of freedom is one. The Horseshoe prior has a similar form as the one-group model, but its prior can lead to a “pseudo-posterior,” which shows the same pattern as the “two-group” model (Bhadra et al., 2017).

Horseshoe prior has already been applied to many scenarios, such as genomic prediction (Pong-Wong and Woolliams, 2014), genome-wide association study (Johndrow et al., 2017), and eQTL mapping (Li et al., 2019). Until now, BayesU (Pong-Wong and Woolliams, 2014) is the only model that uses the Horseshoe prior, and BayesU had similar performance with BayesA and BayesB tested with simulation data. However, the performance of BayesU has not yet been tested with real data. To better separate signals and noise, an extension of the Horseshoe estimator, named Horseshoe+ prior (Bhadra et al., 2017), was proposed. Horseshoe+ introduces one more local parameter with a positive half-Cauchy distribution, which leads a heavier tail than using standard Horseshoe prior. The investigation of using Horseshoe+ prior in Bayesian regression models for genomic prediction could be interesting but has not yet been explored previously. Besides, in variable shrinkage and selection models, hyperparameters is a challenge, and many previous studies have tried to estimate hyperparameter to improve prediction accuracy (Habier et al., 2011; Zhu et al., 2016).

This study’s objectives were to (i) develop two Bayesian methods for genomic prediction based on the global–local prior, which have the flexibility in estimating hyperparameters, and (ii) to test the model performance with simulated and real data for traits with various genetic architectures.

## Materials and Methods

### Statistical Models

In Bayesian regression models, the differences in different models were the prior assumptions on the effects of single-nucleotide polymorphisms (SNPs). All Bayesian multiple regression model can be described as follows:

$$\begin{array}{c} y=\mu +\sum_{k=1}^{m}{\text{x}}_{k}\beta_{k}+\text{e} \end{array},$$

where ***y*** is the vector of pre-corrected phenotypes, **μ** is the overall mean, ***x_k_*** is the vector of genotypes for the *k*th SNP, *m* is the number of SNPs, β_*k*_ is the effect of the *k*th SNP, and ***e*** is a vector of random residuals. The assumptions of the residuals are $\text{e}∼N\left(0,\text{D}\sigma_{e}^{2}\right)$, where $\sigma_{e}^{2}$ is the random residual variance. The ***D*** is an identity matrix when using pre-corrected phenotypes other than de-regressed proofs (DRPs). DRPs were derived from an official estimated breeding value (EBV) with the method that Jairath et al. (1998) suggested. When using DRP as ***y***, ***D*** is a diagonal matrix with diagonal elements calculated as $d_{ii}=\frac{1-r_{i}^{2}}{r_{i}^{2}}$, to account for heterogeneities in $\sigma_{e}^{2}$ due to differences in reliability ($r_{i}^{2}$) of DRP.

In Bayesian inference, a total of 50,000 Markov chain Monte Carlo samples were generated, with the first 20,000 samples discarded as burn-in and every 50th sample of the remaining 30,000 samples saved for inferring posterior statistics. All analyses with Bayesian regression models were conducted using in-house scripts written in Fortran 95 by the first author.

#### BayesU

The BayesU (Pong-Wong and Woolliams, 2014) was developed based on Horseshoe prior (HS). To make it comparable with other methods, the global prior τ was set as a flat prior. A detailed description is given as:

$$\beta_{k}∼N\left(0,\lambda_{k}^{2}\tau^{2}\right),\begin{array}{c} \lambda_{k}∼C^{+}\left(0,1\right) \end{array},and\;\begin{array}{c} \tau ∼flat \end{array}$$

where λ_*k*_ and τ are local and global parameters, respectively. The local parameter λ_*k*_ follows a positive half-Cauchy distribution, which is a special case of student-t distribution where the degree of freedom equals to 1.

#### BayesHP

Compared with HS, its modified version Horseshoe+ (HS+) can form a heavier tailed prior distribution by introducing an additional local parameter with a positive half-Cauchy distribution. Regarding the performance, HS+ can better distinguish the signals and noise than the standard HS (Bhadra et al., 2017). However, Horseshoe+ prior (HS+) (Bhadra et al., 2017) has not yet been applied in Bayesian regression models for genomic prediction. In this study, we proposed a novel BayesHP model based on HS+:

$$\beta_{k}∼N\left(0,\lambda_{k}^{2}\tau^{2}\right),\lambda_{k}∼C^{+}\left(0,\eta_{k}\right),\eta_{k}∼C^{+}\left(0,1\right),$$

$$and\tau ∼C^{+}\left(0,N^{-1}\right)$$

where λ_*k*_ and η_*k*_ are local parameters, and τ is a global parameter following a positive half-Cauchy distribution with scale parameter equals to *N*^−1^. The *N* is the size of training data, as Bhadra et al. (2017) suggested. Compared with HS, HS+ introduces one more layer of local parameter η_*k*_. As described by Makalic and Schmidt (2016) and Makalic et al. (2016) (Appendix), the half-Cauchy distribution can be modeled as a scale mixture of inverse gamma distributions: if $\begin{array}{c} x^{2}∼IG\left(\frac{1}{2},\frac{1}{a}\right) \end{array}$ and $a∼IG\left(\frac{1}{2},\frac{1}{A^{2}}\right),$ then *x*∼*C*^+^(0, *A*). Finally, the distribution of parameters for the revised Horseshoe+ hierarchy is as follows:

$$\beta_{k}∼N\left(0,\lambda_{k}^{2}\tau^{2}\right),\lambda_{k}^{2}∼IG\left(\frac{1}{2},\frac{1}{\theta_{k}}\right),\theta_{k}∼IG\left(\frac{1}{2},\frac{1}{\eta_{k}^{2}}\right),\eta_{k}^{2}$$

$$∼IG\left(\frac{1}{2},\frac{1}{\nu_{k}}\right),\nu_{k}∼IG\left(\frac{1}{2},1\right),\tau^{2}∼IG\left(\frac{1}{2},\frac{1}{\xi }\right),$$

$$and\xi ∼IG\left(\frac{1}{2},N^{2}\right).$$

The conditional posterior distributions of λ_*k*_ and η_*k*_ parameters are inverse-gamma distributions, which makes Gibbs sampling straightforward.

#### BayesHE

In BayesU and BayesHP, the prior of local parameter λ_*k*_ followed a positive half-Cauchy distribution. Both of these two models used a fixed value as the degree of freedom. To increase the flexibility and the suitability of the prediction model, we proposed a new model, named BayesHE, which assumed the local parameter λ_*k*_ to follow a half-t distribution with an unknown degree of freedom υ:

$$\beta_{k}∼N\left(0,\lambda_{k}^{2}\tau^{2}\right),\lambda_{k}∼half-t^{+}\left(\upsilon ,1\right),\tau ∼C^{+}\left(0,N^{-1}\right),$$

andυ∼G(a,b)

By introducing auxiliary variables (Wand et al., 2011), the revised hierarchy is as follows:

$$\beta_{k}∼N\left(0,\lambda_{k}^{2}\tau^{2}\right),\lambda_{k}^{2}∼IG\left(\frac{\upsilon }{2},\frac{\upsilon }{\theta_{k}}\right),\theta_{k}∼IG\left(\frac{1}{2},1\right),$$

$$\tau^{2}∼IG\left(\frac{1}{2},\frac{1}{\xi }\right),\xi ∼IG\left(\frac{1}{2},N^{2}\right),and\upsilon ∼G\left(a,b\right).$$

All parameters, including β_*k*_, $\lambda_{k}^{2}$, θ_*k*_, τ^2^, and *ξ*, had a standard form, except υ. The full conditional distribution of the hyperparameter υ is described as follows:

$$f\left(\upsilon |.\right)\propto f\left(\lambda_{k}^{2}|\upsilon \right)*f\left(\upsilon \right)\propto \prod_{k=1}^{m}\frac{{\left(\frac{\upsilon }{\theta_{k}}\right)}^{\left(\frac{\upsilon }{2}\right)}}{\Gamma_{\left(\frac{\upsilon }{2}\right)}}\lambda_{k}^{2}-\left(\frac{\upsilon }{2}+1\right)\exp$$

$$\left(-\frac{\frac{\upsilon }{\theta_{k}}}{\lambda_{k}^{2}}\right)*\upsilon^{\left(a-1\right)}\exp\left(-b\upsilon \right)\propto \upsilon^{\left(\frac{\upsilon *m}{2}+a-1\right)}*\Gamma {\left(\frac{\upsilon }{2}\right)}^{-m}$$

$$*\exp\left(-\upsilon \left(\frac{1}{2}\sum_{k=1}^{m}\text{ln}\left(\theta_{k}\lambda_{k}^{2}\right)+\sum_{k=1}^{m}\frac{1}{\theta_{k}\lambda_{k}^{2}}+b\right)\right)$$

where *m* is the number of SNPs, Γ_(.)_ is the gamma function, *a* is the shape parameter in gamma distribution, and *b* is the scale parameter of the gamma distribution for υ. In this study, we compared two models with the same *b* (*b* equals to 1) but with different *a*, including BayesHE1 with *a* equals to 4 and BayesHE2 with *a* equals to 5. The hyperparameter was inferred by applying a univariate Metropolis–Hastings sampling (DFMH) process (Yang et al., 2015). The random walk M-H step worked with ζ = log (υ) because υ was inherently positive. The corresponding full conditional distribution of ζ is as follows:

$$f\left(\zeta |ELSE\right)\propto \exp{\left(\zeta \right)}^{\left(\frac{\exp\left(\zeta \right)*m}{2}+a-1\right)}*\Gamma {\left(\frac{\exp\left(\zeta \right)}{2}\right)}^{-m}$$

$$*\exp\left(-\exp\left(\zeta \right)\left[\frac{1}{2}\sum_{k=1}^{m}\text{ln}\left(\theta_{k}\lambda_{k}^{2}\right)+\sum_{k=1}^{m}\frac{1}{\theta_{k}\lambda_{k}^{2}}+b\right]\right)*\exp\left(\zeta \right)$$

$$\propto \exp{\left(\zeta \right)}^{\frac{\exp\left(\zeta \right)*m+2a}{2}}*\Gamma {\left(\frac{\exp\left(\zeta \right)}{2}\right)}^{-m}*$$

$$\exp\left(-\exp\left(\zeta \right)\left[\frac{1}{2}\sum_{k=1}^{m}\text{ln}\left(\theta_{k}\lambda_{k}^{2}\right)+\sum_{k=1}^{m}\frac{1}{\theta_{k}\lambda_{k}^{2}}+b\right]\right)$$

where exp (ζ) is the Jacobian from υ to ζ.

The performance of three Bayesian regression models applying global–local priors, including BayesU, BayesHP, and BayesHE, were further compared with three widely used genomic prediction models, including GBLUP, BayesA, and BayesB.

#### BayesA/BayesB

In BayesB, the prior distribution of β_*k*_ is as follows:

$$\beta_{k}|S_{\beta }^{2},\upsilon ,\pi ∼IID\left\{ \begin{array}{c} 0\;withprobabilitty\pi \\ t\left(0,S_{\beta }^{2},\upsilon \pi \right)\;withprobabilitty1-\pi \end{array}\right.$$

The BayesA model can be considered as a specific case of BayesB, where π = 0. In this study, we set π = 0.95 for BayesB.

#### GBLUP

The GBLUP model is described as follows:

$$\begin{array}{c} y=\mu +\text{Zg}+\text{e} \end{array}$$

where ***y*** is the vector of pre-corrected phenotypes, **μ** is the overall mean, ***Z*** is the design matrix linking genetic value (***g***) to ***y***, and ***e*** is a vector of random residuals. It was assumed that,

$$\text{g}∼N\left(0,\text{G}\sigma_{g}^{2}\right)and\text{e}∼N\left(0,\text{D}\sigma_{e}^{2}\right)$$

where$\sigma_{g}^{2}$ is the additive genetic variance and $\sigma_{e}^{2}$ is the random residual variance. The genomic relationship matrix (***G***) (VanRaden, 2008) was calculated with SNPs:

$$\begin{array}{c} G=\frac{\left(M-P\right)\left(M-P\right){}^{\prime }}{2\sum_{k=1}^{m}p_{k}\left(1-p_{k}\right)} \end{array},$$

where ***M*** is a *n* × *m* matrix with *n* for the number of individuals and *m* for the number of SNPs, *p*_*k*_ is the MAF of *i*th SNP, and ***P*** is the matrix in which the *k*th column elements are 2*p*_*k*_. In this study, GBLUP was implemented using the DMU software (Madsen and Jensen, 2012).

### Datasets

#### Quantitative Trait Locus-Marker-Assisted Selection Data

We used the simulated data from the 15th QTL-marker-assisted selection (MAS) workshop (Elsen et al., 2012) to test model performances. The founder animals consist of 20 sires and 200 dams. For each generation, one sire was mated to 10 dams, and each dam produced 15 offspring. Eight QTLs were simulated across the five chromosomes, with one QTL being quadri-allelic, two linked in phase, two linked in repulsion, one imprinted, and two epistatic. Random residual effects were added to achieve a realized heritability of 0.3. After removing loci without polymorphisms, 7,121 SNPs were retained for analysis. Details on the simulated dataset are in Li et al. (2018).

In each full-sib family, 10 individuals had marker genotypes and phenotype, and the remaining five individuals only had marker genotypes. In total, 2,000 individuals had both genotype and phenotype information, and 1,000 individuals only had genotype information. In this study, only 2,000 individuals with genotypes and phenotypes were used for cross-validation.

#### Cattle Data

For real data analysis in dairy cattle, we collected phenotypic and genomic data from Chinese Holsteins. In total, 7,052 individuals were available for analyses on three milk production traits, including milk yield (MY), fat yield (FY), and protein yield (PY), and on one health traits (somatic cell score, SCS), and 3,530 individuals were available for three type traits including conformation (CONF), feet lag (FL), and mammary system (MS). DRP derived from the official EBV were used as pseudo-phenotypes for genomic prediction. The reliability of DRP for each individual was estimated as $r_{DRP}^{2}=ERC_{i}/\left(ERC_{i}+\lambda \right),$ with $\lambda =\frac{1-h^{2}}{h^{2}}$, where *ERC*_*i*_ refers to the effective record contribution and *h*^2^ refers to the estimated heritability of the trait. On note, effective record contribution (*ERC*_*i*_) was relevant to the reliability (*REL*_*i*_) of the EBV of animal *i* (Přibyl et al., 2013), *ERC*_*i*_ = λ*REL/(1−*RELi*. Animals were genotyped by the Illumina 50K chip. Missing genotypes were imputed with Beagle version 3 (Browning and Browning, 2011). We further removed the SNPs with a minor allele frequency below 0.01 and significantly deviated from Hardy–Weinberg equilibrium (*p* < 10^−6^) and the individuals with call rates lower than 0.90. After quality control, 43,447 SNPs remained for subsequent analyses.

#### Mice Data

For real data analysis in mice, we used the heterogeneous stock mice dataset generated by the Wellcome Trust Centre for Human Genetics^1^. As described by Legarra et al. (2008), the extent of linkage disequilibrium in this population is strong, with an average $r_{LD}^{2}$ among adjacent SNPs being 0.62. To compare the performance of different methods, we selected three traits: growth rate between 6 and 10 weeks of age (GSL), body mass index (BMI), and body length (BL). There were 1,821, 1,814, and 1,901 individuals available for analysis on BL, BMI, and GSL, respectively. In total, 9,098 SNPs were available. A detailed description of the population can be found in Li et al. (2018).

### Cross-Validation and Prediction Accuracy

To assess the prediction accuracy, a 5 × 6 cross-validation (six-fold cross-validation repeated five times) procedure was used, and the results are shown as the mean and standard error for replicates. The performances of all methods were evaluated by examining the accuracy of direct genomic value (DGV) in test data. For QTL-MAS and mice data, Pearson correlation of DGV and phenotype/pre-corrected phenotype was used; for cattle data, the prediction accuracy was further corrected by the average accuracy (square root of reliability) of DRP in test data:

$$acc=\frac{cor\left(DRP,DGV\right)}{\bar{r}}$$

where *cor*(*DRP*,*DGV*) is the Pearson correlation of DRP and DGV of the validation data, and $\bar{r}$ is the average of the square root of the testing data DRP reliabilities.

In addition, the regression of DRP on phenotype,*y*, was used to evaluate the unbiasedness of prediction for all three datasets. The closer the regression coefficient to one, the more unbiased the prediction result.

## Results

In this study, the performance of our newly proposed Bayesian models with global–local priors was compared with GBLUP, BayesA, BayesB (π = 0.95), and BayesU, using the simulated data generated by QTL-MAS, and real data in cattle and mice.

**FIGURE 1:**
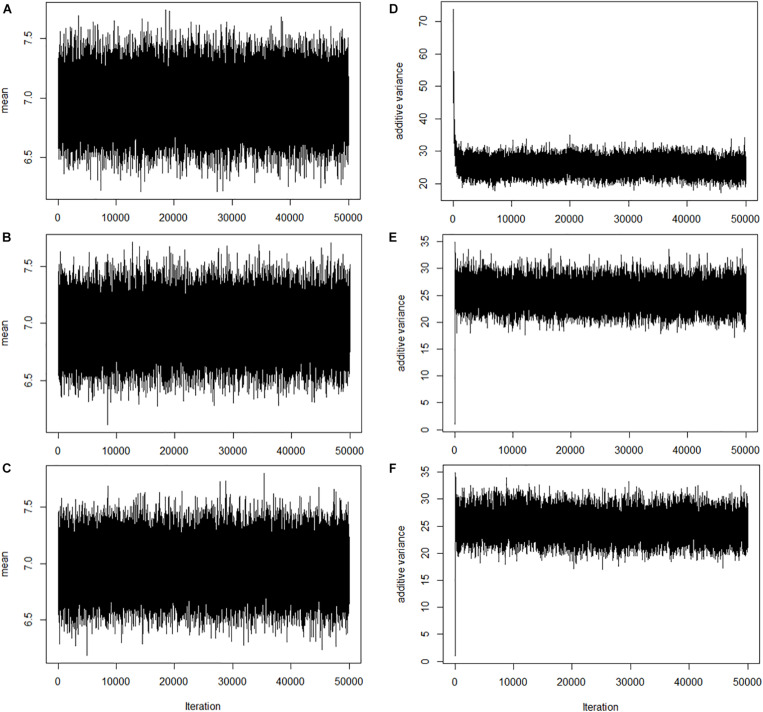
Trace plots of overall mean and additive variance for BayesHP and BayesHE. **(A–C)** Trace plots of overall mean for BayesHP, BayesHE1, and BayesHE2; **(D–F)** Trace plots of additive variance for BayesHP, BayesHE1, and BayesHE2.

To assess the convergence of Markov chain Monte Carlo, trace plots of the overall mean (**μ**) and additive variance [$V_{g}=\text{var}\left(\sum_{k=1}^{m}x_{k}\beta_{k}\right)$] are shown in Figure 1. Also, the trace plots suggested that parameters mixed well. However, additive variance from BayesHE (Figures 1E,F) converges faster than BayesHP (Figure 1D).

### Quantitative Trait Locus-Marker-Assisted Selection Data

Table 1 shows the prediction accuracies and bias for all models based on the 15th QTL-MAS workshop dataset. Regarding the prediction accuracy, Bayesian regression models with global–local priors, such as BayesU (0.506), BayesHP (0.505), BayesHE1 (0.505), and BayesHE2 (0.505), outperformed all other methods. The prediction biases of the seven methods were similar and close to one.

**TABLE 1:** Prediction accuracies and biases of DGVs of test dataset from 15th QTL-MAS data using six-fold cross-validation with five replications.

	Accuracy	Bias
GBLUP	0.456±0.002	1.010±0.004
BayesA	0.474±0.009	0.924±0.008
BayesB	0.475±0.013	0.925±0.004
BayesU	0.506±0.002	0.996±0.004
BayesHP	0.505±0.002	1.000±0.005
BayesHE1	0.505±0.002	1.005±0.006
BayesHE2	0.505±0.002	1.003±0.005

*DGVs, direct genomic value; and the mean and standard errors are shown in the table.*

### Cattle Data

The prediction accuracies of seven traits in the Chinese Holstein population that the mean $r_{LD}^{2}$ of adjacent SNP pairs ranged from 0.16 to 0.24 (Zhou et al., 2013) are shown in Table 2. Generally, BayesHE with two modalities (e.g., BayesHE1 and BayesHE2) on hyperparameters achieved optimal or suboptimal prediction accuracy for all of the seven traits.

**TABLE 2:** Prediction accuracies of seven traits of dairy cattle using six-fold cross-validation with five replications.

	MY	FY	PY	SCS	CONF	FL	MS
GBLUP	0.451±0.002	0.410±0.002	0.435±0.001	0.356±0.002	0.480±0.003	0.676±0.004	0.428±0.006
BayesA	0.467±0.002	0.425±0.002	0.433±0.001	0.365±0.002	0.478±0.003	0.677±0.004	0.425±0.006
BayesB	0.455±0.003	0.401±0.002	0.421±0.002	0.345±0.002	0.380±0.037	0.656±0.005	0.399±0.006
BayesU	0.463±0.003	0.415±0.002	0.420±0.002	0.346±0.003	0.447±0.007	0.664±0.007	0.404±0.008
BayesHP	0.459±0.003	0.410±0.002	0.414±0.003	0.341±0.003	0.440±0.009	0.660±0.007	0.401±0.008
BayesHE1	0.473±0.003	0.427±0.002	0.435±0.001	0.365±0.002	0.478±0.003	0.674±0.004	0.426±0.006
BayesHE2	0.473±0.003	0.427±0.002	0.434±0.001	0.365±0.002	0.478±0.003	0.674±0.004	0.427±0.005

*MY, milk yield; FY, fat yield; PY, protein yield; SCS, somatic cell score, CONF, conformation; FL, feet lag; MS, mammary system; and the mean and standard errors are shown in the table.*

For milk production traits, Bayesian regression models with global–local priors had a better performance compared with GBLUP, BayesA, or BayesB, especially for MY. For example, the prediction accuracy of BayesHE1 was 0.473, which increased approximately 2.2% than GBLUP. Also, BayesHE1 had similar prediction accuracy than BayesHE2. However, for SCS, BayesHP achieved the lowest prediction accuracy (0.341), and BayesHE and BayesA had similar prediction accuracy (0.365).

For type traits, BayesB, BayesU, and BayesHP did not perform well, and GBLUP, BayesA, and BayesHE had similar prediction accuracy. Notably, BayesHE2 performed similarly to BayesHE1. Although BayesHE did not perform the best, the prediction accuracy was very close to that of the best model. For example, the prediction accuracy for MS from BayesHE2 was 0.427, which was only slightly lower than the highest prediction accuracy achieved by GBLUP (0.428).

The biases of prediction for the seven traits are shown in Table 3. For MY, FY, PY, and SCS, BayesHE1 achieved the least bias of prediction. The performance of BayesHE2, regarding bias, was very close to that of BayesHE1. For FL, BayesA was the most unbiased model, and BayesHE2 was the second best.

**TABLE 3:** Prediction biases of seven traits of dairy cattle data using six-fold cross-validation with five replications.

	MY	FY	PY	SCS	CONF	FL	MS
GBLUP	0.865±0.004	0.817±0.003	0.814±0.002	0.807±0.006	0.803±0.006	0.829±0.007	0.826±0.014
BayesA	0.877±0.005	0.807±0.003	0.807±0.002	0.812±0.005	0.798±0.006	0.837±0.007	0.809±0.014
BayesB	0.824±0.006	0.755±0.003	0.750±0.004	0.756±0.006	0.763±0.021	0.775±0.006	0.740±0.014
BayesU	0.887±0.007	0.828±0.004	0.816±0.007	0.812±0.008	0.749±0.019	0.812±0.011	0.786±0.020
BayesHP	0.889±0.007	0.829±0.005	0.816±0.007	0.815±0.007	0.735±0.022	0.805±0.011	0.772±0.019
BayesHE1	0.906±0.007	0.835±0.003	0.821±0.002	0.821±0.006	0.805±0.007	0.833±0.007	0.831±0.015
BayesHE2	0.905±0.008	0.834±0.003	0.819±0.002	0.820±0.006	0.807±0.007	0.834±0.007	0.833±0.014

*MY, milk yield; FY, fat yield; PY, protein yield; SCS, somatic cell score, CONF, conformation; FL, feet lag; MS, mammary system; and the mean and standard errors are shown in the table.*

### Mice Data

The prediction accuracies of three mice traits with different methods are shown in Table 4. In the analysis of mice data, there were two kinds of traits: one growth trait (GSL) and two type traits (BL and BMI). For all three traits, BayesHP performed the worst. For example, the prediction accuracy of BL from BayesHP was 0. 253, but accuracies from other methods were greater than 0.260. However, GBLUP, BayesA, and BayesHE had similar prediction accuracies.

**TABLE 4:** Prediction accuracies of mice data using six-fold cross-validation with five replications.

	BL	BMI	GSL
GBLUP	0.272±0.002	0.226±0.002	0.386±0.003
BayesA	0.275±0.002	0.227±0.002	0.385±0.003
BayesB	0.268±0.001	0.217±0.002	0.374±0.003
BayesU	0.261±0.002	0.220±0.003	0.374±0.003
BayesHP	0.253±0.003	0.214±0.003	0.368±0.004
BayesHE1	0.274±0.002	0.229±0.002	0.386±0.003
BayesHE2	0.272±0.001	0.227±0.002	0.386±0.003

*BL, body length; BMI, body mass index; GSL, growth slope; and the mean and standard errors are shown in the table.*

Table 5 shows the prediction bias. The regression coefficients were close to the unity for all traits using all models, which indicated unbiasedness of the predictions. Nevertheless, there were still some slight differences. For example, the unbiasedness of BayesB was slightly lower than other models for all traits.

**TABLE 5:** Prediction biases of mice data using six-fold cross-validation with five replications.

	BL	BMI	GSL
GBLUP	0.988±0.012	1.023±0.022	1.004±0.010
BayesA	0.995±0.009	0.988±0.015	0.988±0.008
BayesB	0.948±0.004	0.929±0.017	0.959±0.007
BayesU	0.972±0.007	0.999±0.036	0.996±0.012
BayesHP	0.981±0.011	1.032±0.039	1.002±0.015
BayesHE1	1.006±0.011	1.024±0.021	1.004±0.009
BayesHE2	1.003±0.009	1.016±0.021	1.005±0.008

*BL, body length; BMI, body mass index; GSL, growth slope; and the mean and standard errors are shown in the table.*

## Discussion

In Bayesian regression models, the differences among methods are the assumptions on the genetic marker effects. Because of the flexibility of the Bayesian method, it has attracted increasing attention. In classical one-group models, both signals and noises were assumed to follow one single continuous prior distribution, where the effects of some markers were shrunk toward zero, relying on the posterior distribution. For the two-group model or the spike-and-slab model, the prior regarding the proportion of genetic markers being signal usually impact its performance in genomic prediction. Some two-group models, such as BayesCπ (Habier et al., 2011), have been developed to estimate the proportion of non-zero effect markers based on both prior and the analyzed data. However, there is a poor convergence and mixing in some situations. The global–local prior, which can shrink signals and noises through local and global parameters, seems to be a good alternative, theoretically. Global–local priors is a kind of continuous shrinkage prior, which can adaptively shrink noise to zero while leaving the large data-supported signal unshrunk (Ge et al., 2019).

The model’s performance depended on the genetic architecture of the trait. The results of simulation indicated that models based on global–local priors, e.g., BayesU, BayesHP, and BayesHE, performed better in traits with higher heritability (i.e., in this study, heritability is 0.3) and fewer QTL. In real data, BayesHE can achieve optimal or suboptimal performance; however, BayesHP performed better only for production traits. Our results suggested that auto-estimate the degree of freedom (e.g., BayesHE) would be a better choice other than increasing the layers of the local parameter (e.g., BayesHP).

The Bayesian models with the assumption more in line with the real distribution of marker effects will result in more accurate predictions. The Bayesian model shrinks the effect of noise markers toward zero and thus increases the prediction accuracy. However, in genomic prediction, markers are not simply signal or noise due to the existence of linkage disequilibrium. It is reasonable that for some traits, GBLUP will achieve better prediction accuracy. For example, GBLUP performed better for type traits (e.g., CONF, FL, and MS) than BayesB, BayesU, and BayesHP, as shown in our results. Notably, in genome-wide association study, regardless of dairy cattle (Wu et al., 2013) or beef cattle (Vallée et al., 2016), there are few significant signals for type traits, suggesting that most genetic variants have similar medium or small effects on the traits. Therefore, it is reasonable why GBLUP had a better performance for type traits.

Many previous studies have suggested that using hyperparameters is likely to improve classical methods (Habier et al., 2011; Yang and Tempelman, 2012; Zhu et al., 2016). In our study, we assumed that the local parameter, λ_*k*_, followed a half-t distribution (BayesHE) with an unknown degree of freedom instead of half-Cauchy (BayesU). By introducing auxiliary variables (Wand et al., 2011), half-t distribution was translated into a scale mixture of the inverse gamma distribution. In BayesHE, $\lambda_{k}^{2}$ was assumed to follow an inverse gamma distribution $IG\left(\frac{\upsilon }{2},\frac{\upsilon }{\theta_{k}}\right)$, which led to an assumption of student-t distribution for marker effects (Wand et al., 2011). The use of unknown shape parameter is similar to the study of Zhu et al. (2016), but the difference is that there is a global parameter τ^2^ in BayesHE model. Besides, in studies of Habier et al. (2011) and Zhu et al. (2016), they set a gamma distribution *G*(1, 1) for the scale parameter. In our study, the scale parameter θ_*k*_ was assumed to follow an inverse gamma distribution, $\theta_{k}∼IG\left(\frac{1}{2},1\right)$. This inspired the authors that the shape parameter of inverse gamma distribution that θ_*k*_ followed can also be set as a variable other than a constant.

Horseshoe-like prior with “U” type shrinkage pattern means strong distinguishment of single and noise. According to the results of QTL detection (Wu et al., 2013; Vallée et al., 2016), Horseshoe-like prior with “U” type may be suitable for genomic prediction of the traits affected by many QTLs with large effect. In our study, we assumed an unknown hyperparameter for the distribution of local parameters, which increased the model flexibility and, therefore, more adaptable to traits with different genetic architectures. The possibility to fit a suitable hyperparameter for the global parameter has been proposed by Armagan et al. (2011), where they assumed global parameter followed a gamma distribution with different shape parameter or just set as a constant value. Their study suggested that the changes of hyperparameters of distributions that local parameters followed and the value of global parameter led to different shrinkage patterns on covariates. In the study of Piironen and Vehtari (2016), the global parameter τ was set as a constant value or followed a normal or half-Cauchy distribution, and they recommended τ half-Cauchy distribution, $\tau ∼C^{+}\left(0,\tau_{0}^{2}\right)$, where the scale parameter $\tau_{0}^{2}$ is relevant to the effective number of variables with non-zero effects. In the global–local prior method, the marker variances were shaped by global and local parameters simultaneously. The global parameter,τ, usually causes the marker effect to approach zero, whereas the local parameter, λ_*k*_, allows marker variance to escape the shrinkage when that marker has a large effect. In future research, more investigation on choosing the type of distribution for global parameters could be interesting.

The limitation of our study is that it mostly focused on statistical perspectives and lack of consideration of the biological information. With time, an increasing amount of biological information affecting complex traits will be detected. It is reasonable to integrate these genomic features into the prediction model, and then, how to effectively utilize these genomic features is worth exploring. The BayesRC model proposed by MacLeod et al. (2016) divides the genome into three major categories: trait-associated genes, regular regions, and other variations. However, there are some challenges in utilizing biological information because of the dynamics in biological processes.

## Conclusion

Our results showed that BayesHE could achieve optimal or suboptimal performance. Compared with other methods, such as GBLUP and BayesA, BayesHP did not perform better. With the automatic estimation of hyperparameters, BayesHE was more flexible than BayesU and BayesHP for the adaptation to a wider range of traits. This suggested that auto-estimate the degree of freedom (e.g., BayesHE) would be a better choice other than increasing the layers of a local parameter (e.g., BayesHP).

## Data Availability Statement

The data analyzed in this study is subject to the following licenses/restrictions: All information supporting the results is included in the text and tables. The dataset of cattle is not publicly available due to commercial restrictions. Requests to access these datasets should be directed to the corresponding author (SZ).

## Ethics Statement

The animal study was reviewed and approved by the Institutional of Animal Care and Use Committee (IACUC), China Agricultural University. Written informed consent was obtained from the owners for the participation of their animals in this study.

## Author Contributions

SS conceived the study, wrote the program, analyzed the data, and drafted the manuscript. SZ and XD conceived the study and supervised the project. XL and LF participated in the design and helped to draft the manuscript. AL helped to analyze the data and revised the manuscript. GS revised the manuscript. YZ and BL helped to draft the manuscript. All authors read and approved the final manuscript.

## Conflict of Interest

The authors declare that the research was conducted in the absence of any commercial or financial relationships that could be construed as a potential conflict of interest.

## Footnotes

1 http://gscan.well.ox.ac.uk/

## Appendix

### Appendix A: Gibbs Sampler for SNP Effect β_*k*_

The full conditional distribution of β_*k*_ for BayesHP and BayesHE can be written as,

$$f\left(\beta_{k}|ELSE\right)\propto f\left(y|\mu ,\beta ,\sigma_{e}^{2}\right)*f\left(\beta_{k}|\tau^{2},\lambda_{k}^{2}\right)\propto \exp\left[-\frac{{\left(\text{y}-\mu -\sum_{k=1}^{m}{\text{x}}_{k}\beta_{k}\right)}^{\prime }{\text{R}}^{-1}\left(\text{y}-\mu -\sum_{k=1}^{m}{\text{x}}_{k}\beta_{k}\right)}{2\sigma_{e}^{2}}\right]*\exp\left[-\frac{\beta_{k}^{2}}{2\lambda_{k}^{2}\tau^{2}}\right]\propto \text{exp}\left[-\frac{1}{2}\frac{{\left(\beta_{k}-{\hat{\beta }}_{k}\right)}^{2}}{\sigma_{e}^{2}/c_{k}}\right]\propto \text{N}\left({\hat{\beta }}_{k},\sigma_{e}^{2}/c_{k}\right)$$

where ${\hat{\beta }}_{k}=\frac{x_{k}^{\prime }R^{-1}w}{c_{k}}$, $w=\text{y}-\mu -\sum_{j\neq k}^{m}{\text{x}}_{j}\beta_{j}$ and $c_{k}=\left(x_{k}^{\prime }R^{-1}x_{k}+\frac{\sigma_{e}^{2}}{\lambda_{k}^{2}\tau {}^{2}}\right)$.

### Appendix B: Gibbs Sampler for $\lambda_{k}^{2}$

The full conditional distribution of $\lambda_{k}^{2}$ can be written as Makalic et al. (2016),

$$f\left(\lambda_{k}^{2}|ELSE\right)\propto f\left(\beta_{k}|\tau^{2},\lambda_{k}^{2}\right)*f\left(\lambda_{k}^{2}|\theta_{k},v\right)\propto {\left(2\pi \lambda_{k}^{2}\tau^{2}\right)}^{-\frac{1}{2}}\exp\left[-\frac{\beta_{k}^{2}}{2\lambda_{k}^{2}\tau^{2}}\right]*\lambda_{k}^{2-\left(\frac{v}{2}+1\right)}\text{exp}\left(-\frac{\frac{v}{\theta_{k}}}{\lambda_{k}^{2}}\right)\propto \lambda_{k}^{2-\left(\frac{v}{2}+\frac{1}{2}+1\right)}\text{exp}\left[-\frac{1}{\lambda_{k}^{2}}\left(\frac{\beta_{k}^{2}}{2\tau^{2}}+\frac{v}{\theta_{k}}\right)\right]\propto IG\left(\frac{v}{2}+\frac{1}{2},\frac{\beta_{k}^{2}}{2\tau^{2}}+\frac{v}{\theta_{k}}\right)$$

Notably, in BayesHP, *v* equals to one.

### Appendix C: Gibbs Sampler for θ_*k*_

The full conditional distribution of θ_*k*_ can be written as Makalic et al. (2016),

$$f\left(\theta_{k}|ELSE\right)\propto f\left(\lambda_{k}^{2}|\theta_{k},v\right)*f\left(\theta_{k}\right)\propto {\left(\frac{p}{\theta_{k}}\right)}^{\left(\frac{p}{2}\right)}*\exp\left(-\frac{\frac{v}{\theta_{k}}}{\lambda_{k}^{2}}\right)*\theta_{k}^{-\left(\frac{1}{2}+1\right)}\text{exp}\left(-\frac{1}{\theta_{k}}\right)\propto \theta_{k}^{-\left(\frac{v}{2}+\frac{1}{2}+1\right)}\text{exp}\left(-\frac{1}{\theta_{k}}\left(1+\frac{v}{\lambda_{k}^{2}}\right)\right)\propto IG\left(\frac{v}{2}+\frac{1}{2},1+\frac{v}{\lambda_{k}^{2}}\right)$$

Similarly, in BayesHP, *v* equals to one.

### Appendix D: Gibbs Sampler for $\eta_{k}^{2}$

The full conditional distribution of $\eta_{k}^{2}$ can be written as Makalic et al. (2016),

$$f\left(\eta_{k}^{2}|ELSE\right)\propto f\left(\theta_{k}|\eta_{k}^{2}\right)*f\left(\eta_{k}^{2}|\nu_{k}\right)\propto {\left(\eta_{k}^{2}\right)}^{-\frac{1}{2}}\exp\left(-\frac{{\left(\eta_{k}^{2}\right)}^{-1}}{\theta_{k}}\right)*{\left(\eta_{k}^{2}\right)}^{-\left(\frac{1}{2}+1\right)}\exp\left(-\frac{\nu_{k}^{-1}}{\eta_{k}^{2}}\right)\propto \eta_{k}^{2-\left(1+1\right)}*\exp\left(-\frac{1}{\eta_{k}^{2}}\left(\frac{1}{\nu_{k}}+\frac{1}{\theta_{k}}\right)\right)\propto IG\left(1,\frac{1}{\nu_{k}}+\frac{1}{\theta_{k}}\right)$$

### Appendix E: Gibbs Sampler for ν_*k*_

The full conditional distribution of ν_*k*_ can be written as Makalic et al. (2016),

(1) $$f\left(\nu_{k}|ELSE\right)\propto f\left(\eta_{k}^{2}|\nu_{k}\right)*f\left(\nu_{k}\right)\propto \nu_{k}^{-\frac{1}{2}}\exp\left(-\frac{\nu_{k}^{-1}}{\eta_{k}^{2}}\right)*\nu_{k}^{-\left(\frac{1}{2}+1\right)}\exp\left(-\frac{1}{\nu_{k}}\right)\propto \nu_{k}^{-\left(1+1\right)}\exp\left(-\frac{1}{\nu_{k}}\left(1+\frac{1}{\eta_{k}^{2}}\right)\right)\propto IG\left(1,1+\frac{1}{\left(\eta_{k}^{2}\right)}\right)$$

### Appendix F: Gibbs Sampler for τ^2^

The full conditional distribution of τ^2^ can be written as Makalic et al. (2016),

$$f\left(\tau^{2}|ELSE\right)\propto f\left(\tau^{2}|\xi \right)*\prod_{k=1}^{m}f\left(\beta_{k}|\tau^{2},\lambda_{k}^{2}\right)\propto {\left(\tau^{2}\right)}^{-\left(\frac{1}{2}+1\right)}\exp\left(-\frac{\xi^{-1}}{\tau^{2}}\right)*\prod_{k=1}^{m}{\left(2\pi \lambda_{k}^{2}\tau^{2}\right)}^{-\frac{1}{2}}\exp\left(-\frac{\beta_{k}^{2}}{2\lambda_{k}^{2}\tau^{2}}\right)\propto \tau^{2-\left(\frac{k+1}{2}+1\right)}\text{exp}\left[-\frac{1}{\tau^{2}}\left(\frac{1}{\xi }+\sum \frac{\beta_{k}^{2}}{2\lambda_{k}^{2}}\right)\right]\propto IG\left(\frac{k+1}{2},\frac{1}{\xi }+\sum \frac{\beta_{k}^{2}}{2\lambda_{k}^{2}}\right)$$

### Appendix G: Gibbs Sampler for ξ

The full conditional distribution of ξ can be written as Makalic et al. (2016),

$$f\left(\xi |ELSE\right)\propto f\left(\tau^{2}|\xi \right)*f\left(\xi \right)\propto \xi^{-\frac{1}{2}}\exp\left(-\frac{\xi^{-1}}{\tau^{2}}\right)*\xi^{-\left(\frac{1}{2}+1\right)}\exp\left(-\frac{N^{2}}{\xi }\right)\propto \xi^{-\left(1+1\right)}*\exp\left[-\left(\frac{1}{\tau^{2}}+N^{2}\right)\frac{1}{\xi }\right]\propto IG\left(1,\frac{1}{\tau^{2}}+N^{2}\right)$$
